# Human DC-SIGN and CD23 do not interact with human IgG

**DOI:** 10.1038/s41598-019-46484-2

**Published:** 2019-07-10

**Authors:** A. Robin Temming, Gillian Dekkers, Fleur S. van de Bovenkamp, H. Rosina Plomp, Arthur E. H. Bentlage, Zoltán Szittner, Ninotska I. L. Derksen, Manfred Wuhrer, Theo Rispens, Gestur Vidarsson

**Affiliations:** 1 Department Experimental Immunohematology, Sanquin Research and Landsteiner Laboratory, Academic Medical Centre, University of Amsterdam, Amsterdam, The Netherlands; 2 Department Immunopathology, Sanquin Research and Landsteiner Laboratory, Academic Medical Centre, University of Amsterdam, Amsterdam, The Netherlands; 3 Center for Proteomics and Metabolomics, Leiden University Medical Center, Leiden, The Netherlands

**Keywords:** Autoimmunity, Inflammatory diseases, Glycosylation

## Abstract

The precise mechanisms underlying anti-inflammatory effects of intravenous immunoglobulin (IVIg) therapies remain elusive. The sialylated IgG fraction within IVIg has been shown to be therapeutically more active in mouse models. Functionally, it has been suggested that IgG undergoes conformational changes upon Fc-sialylation which sterically impede binding to conventional FcγRs, but simultaneously allow binding to human DC-SIGN (SIGN-R1 in mice) and also CD23. These latter C-type lectins have been proposed responsible for the immunomodulatory effects in mouse models. However, there is conflicting evidence supporting direct interactions between sialylated human IgG and CD23/DC-SIGN. While cells expressing human CD23 and DC-SIGN in their native configuration bound their natural ligands IgE and ICAM-3, respectively, no IgG binding was observed, regardless of Fc-glycan sialylation in any context (with or without bisection and/or fucosylation) or presence of sialylated Fab-glycans. This was tested by both by FACS and a novel cellular Surface Plasmon Resonance imaging (cSPRi) approach allowing for monitoring low-affinity but high-avidity interactions. In summary, we find no evidence for human CD23 or DC-SIGN being *bona fide* receptors to human IgG, regardless of IgG Fc- or Fab-glycosylation status. However, these results do not exclude the possibility that either IgG glycosylation or C-type lectins affect IVIg therapies.

## Introduction

Human immunoglobulin G (IgG) molecules contain a conserved *N*-linked glycan in their crystallizable fragment (Fc) at the asparagine residue at position 297 (N297). In addition, approximately 15% of total IgGs have *N-*glycans in the antigen binding fragment (Fab) in the antigen binding part (VH and VL domains)^1,2^. These glycans have a biantennary core structure compose d of mannose and *N*-acetylglucosamine (GlcNAc) residues which can be extended with other sugar moieties such as fucose, galactose and sialic acid. Fc-glycans are required for the open conformation of the CH2 region of the IgG Fc domain to facilitate binding of Fc gamma receptors (FcγRs)^3^. Subtle changes in Fc-glycan composition influence the affinity of IgG for FcγRs and effector functions^4^. For example, it is well established that afucosylated IgG has an enhanced binding affinity to FcγRIIIa and FcγRIIIb^4–8^. Recently, it has become apparent that galactosylation, which has less effect on binding affinity of fucosylated IgG to FcγR, further enhances binding affinity of afucosylated IgG to FcγRIIIa/b^7,9^. Notably, the elevated binding of these IgG glycoforms to members of the FcγRIII family (FcγRIV in mice) seems to be related to the fact that only these receptors have an *N*-linked glycan at position 162 that also directly interacts with the IgG Fc-glycan, causing favorable binding to afucosylated IgG^10–12^. Irrespective of the fucosylation status, IgG Fc-galactosylation and -sialylation, increases the binding of IgG to C1q and thereby affects classical complement activation^7,13^. Therefore, IgG glycosylation encodes for an important immunomodulatory ability.

Because of its anti-inflammatory effect, intravenous immunoglobulin (IVIg) therapy is used to treat a wide range of patients with autoimmune diseases and other inflammatory conditions. The precise mechanisms underlying this treatment still remain to be elucidated. The current ideas are that several mutually nonexclusive mechanisms exist, IgG Fab- and/or Fc-mediated, that are responsible for its clinical actions^14^. Because of the immunomodulatory potential, IgG glycosylation has been suggested to be an additional factor responsible for the effectiveness of IVIg treatment^15–20^. During the last decade, a considerable amount of prominent work has been put forward hinting at an alternative mechanism^17,18,21–23^. One suggested mechanism is that the IVIg fraction with sialylated Fc (approximately 16% of total IVIg^24^) is responsible for the anti-inflammatory effect via the C-type lectin DC-SIGN (dendritic cell-specific ICAM-3 grabbing non-integrin), which has been designated by some as a type II FcR^25^. Another member of this Type II FcR family is the C-type lectin CD23 (low affinity receptor for IgE Fc). In the proposed mechanism, these homologous receptors (both located on chromosome 19^26^) can both bind the IgG CH2-CH3 interphase, in a similar fashion as CD23 binds to IgE^23^. IgG binding to DC-SIGN, found on regulatory macrophages and dendritic cells, has been reported to result in the release of regulatory cytokines and subsequently upregulation of the inhibitory FcγRIIb on effector macrophages, causing an increased threshold for inflammation^17,19,25^. IgG binding to CD23, found on mature B-cells, has also been reported to cause upregulation of FcγRIIb, which increases the threshold for B-cell receptor signaling and affects Ig-affinity maturation^27,28^.

According to this model, CD23 and DC-SIGN only bind to IgG, in a calcium-dependent manner, when the IgG Fc-glycan is α2,6-sialylated. Upon sialylation, the Fc domain was hypothesized to undergo a conformational change from an “open” to a “closed” state^23^. However, follow-up work has suggested that these structural changes are minimal and confined to the CE loop in the upper CH2 region^29,30^. In addition, direct evidence for the interaction between IgG and CD23/DC-SIGN consists of two cell-based ELISA assays demonstrating that CD23/DC-SIGN-expressing Chinese hamster ovary (CHO) cells preferentially bind to sialylated human IgG Fc derived from IVIg or recombinant human IgG1^18,23^. Predicted interactions between crystal structures and the effects that can be observed in *in vitro* and *in vivo* tests provide further indirect evidence^18,21,25,27,28,31,32^. One of these *in vivo* studies used a transgenic mouse model in which a SIGN-R1 (specific ICAM-grabbing non-integrin R1) knock-out resulted in an abolishment of the anti-inflammatory response after administration of sialylated IgG^21^. Transgenic expression of human DC-SIGN in these SIGN-R1-deficient mice resulted in restoration of this immunomodulatory response^27,28^.

However, a considerable amount of contradictive studies emerged, mostly showing that DC-SIGN is not involved and/or sialylated IgG is not a stronger anti-inflammatory substance in several *in vitro* functional assays^15,20,33,34^ and/or in mouse models^35–37^. Some studies have suggested that it is not sialylation in the Fc but rather sialylation in the Fab domain that may exert certain anti-inflammatory effects^20^, while others found no additional effect of Fab-sialylated IVIg^36^. One group demonstrated that binding of recombinant DC-SIGN tetramers to hexameric IgG Fc-based scaffolds does not depend on sialylation, but rather on high-mannose levels on the *N*-linked glycan^38^, which was also found in the glycan binding assays performed by Guo *et al*.^39^. Interestingly, Yu *et al*. demonstrated in an ELISA-based study that well-characterized sialylated IgG Fc fragments do not bind directly to human DC-SIGN^40^.

For most of these binding studies, either a well-defined set of glyco-engineered IgG was lacking^18,23^, or the human CD23-IgG interaction was not studied^40^. The aforementioned controversies, extensively reviewed elsewhere^41,42^, need to be settled.

To study this in more detail, we extensively investigated the direct binding between native human CD23/DC-SIGN and a well-characterized set of human IgG glycoforms. In addition to flow cytometry analysis, we also used a biosensor platform allowing us to study low affinity and avidity interactions for both receptors CD23 and DC-SIGN expressed in its native configuration on cells. Furthermore, the context of the IgG α2,6-sialylation was studied, by including both Fab-sialylated IgG and altering the naturally-occurring variable glycan adducts in the sialylated Fc-glycan, in any possible combination.

## Results

### Generation of human IgG1 glycoforms

**Figure 1:**
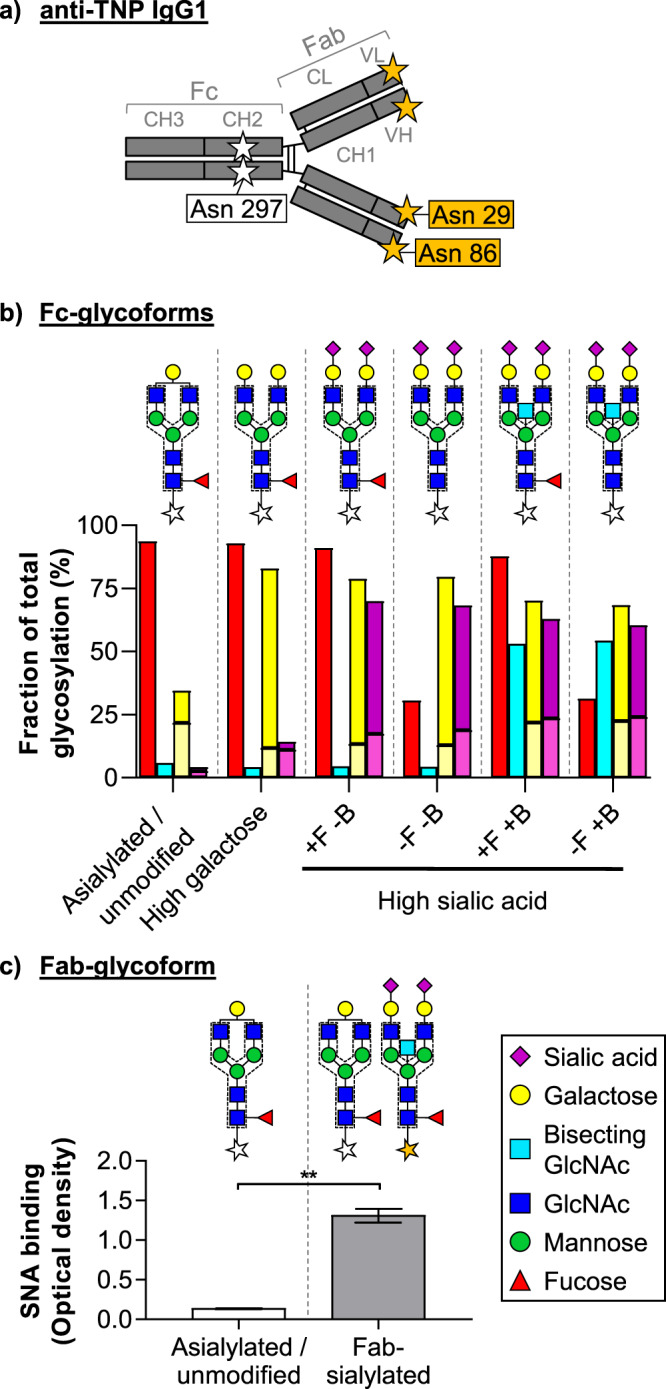
Generation of glyco-engineered human IgG1. (**a**) Schematic representation of glyco-engineered IgG1 antibodies generated in this study with their biantennary glycan on asparagine (Asn) 297 (white star) in the Fc CH2 domain and, for the Fab-glycoform, on Asn 29 and Asn 86 (orange stars) in the VH and VL domain, respectively. (**b**) Bar graph summarizing Fc-glycoform *N*-glycan profile of variable glycan end groups (fucose (+/−F), in red; bisecting GlcNAc (+/−B) in cyan; mono-/di-galactose in pale/dark yellow; mono-/di-sialic acid in pale/dark purple) determined by mass spectrometry as percentages (%) of total glycosylation. *N*-glycan structures are schematically represented; galactose (yellow circle); mannose (green circle); *N*-acetylglucosamine (blue square); bisecting *N*-acetylglucosamine (cyan square); fucose (red triangle); sialic acid (purple diamond). (**c**) Bar graph showing the level of sialic acid of Fab-glycosylated anti-TNP as determined by *Sambucus nigra* agglutinin (SNA)-lectin ELISA with high sialic acid Fab-glycoform (grey bar) to asialylated (unmodified) IgG1 without a Fab-glycan (white bar). Data are representative of three independent experiments (in triplicate) showing mean ± standard error of the mean (s.e.m.). Statistical analysis was performed by a Two-tailed paired t-test (**P < 0.01).

In order to test whether IgG glycosylation determines the binding capacity to the proposed IgG Fc receptors DC-SIGN and CD23, we first generated a defined set of glyco-engineered recombinant human IgG1 variants (Fig. 1)^7,43^. Our choice for IgG1 was based on the fact that it comprises the largest fraction in IVIg (approximately 60%)^44^ and because studies demonstrating direct binding of sialylated IgG to CD23/DC-SIGN all used Fc fragments which originated either from IVIg or recombinant human IgG1^18,23^. Also the immunomodulatory effects in mouse models that have been shown for sialylated IgG from IVIg^18,22,45^ could be fully recapitulated by sialylated recombinant human IgG1^27,28,46,47^. The produced IgG Fc-glycoforms (asialylated/unmodified; galactosylated; sialylated with (+) or without (−) fucose (F) or bisecting GlcNAc (B)) were characterized by tandem liquid chromatography and mass spectrometry (Fig. 1b and Supplemental Fig. S1), and show the expected glycan features. Levels of di-galactosylation and di-sialylation of those IgG glycoforms enriched in galactose and sialic acid, were highly elevated above that of mono-galactosylated and mono-sialylated IgG species (Fig. 1b and Supplemental Fig. S1). In addition, IgG1 with Fab glycosylation sites (known to have high level of α2,6-sialic acid)^36,48^ were produced and sialylation levels were verified by performing a *Sambucus nigra* agglutinin (SNA)-ELISA assay (Fig. 1c).

### Human CD23 and DC-SIGN expressed on human embryonic kidney cells recognize natural ligands

**Figure 2:**
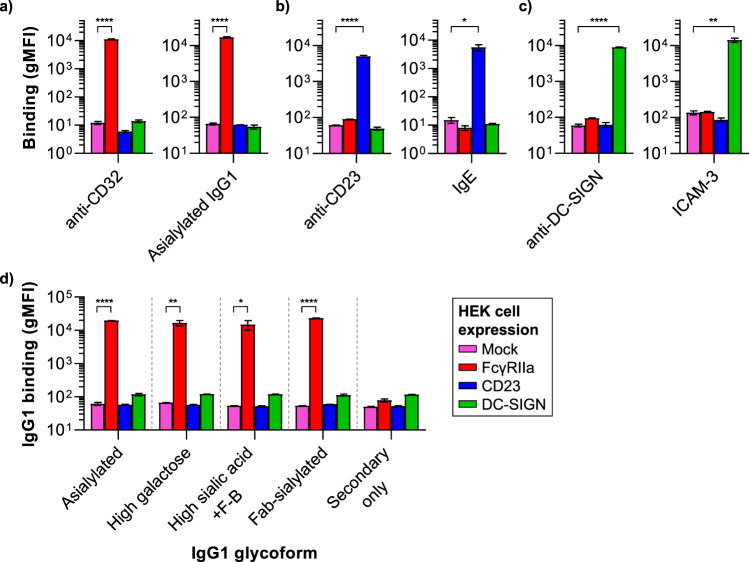
CD23 and DC-SIGN are functionally expressed on transfected HEK Freestyle cells, but do not bind human IgG1, regardless of sialylation status. (**a**–**c**) HEK Freestyle cells were transfected with human FcγRIIa (CD32, red), CD23 (blue), DC-SIGN (green), or an empty vector (Mock, pink) and tested for binding to anti-CD32, anti-CD23. or anti-DC-SIGN, or their natural ligands at 37 °C: asialylated IgG1, IgE, or ICAM-3, respectively. (**d**) Binding of different soluble IgG1 glycoforms (10 μg/ml) to HEK CD23, DC-SIGN, FcγRIIa and Mock transfected cells at 37 °C. Data are representative of three independent experiments (in duplicate) and shown as mean values of the geometric mean fluorescence intensity (gMFI ± s.e.m.) with the background signals subtracted. Significant differences compared to HEK Mock signals were determined by One-way ANOVA with Dunnett’s multiple comparisons test (*P < 0.05, **P < 0.01, ****P < 0.0001).

Human CD23 and DC-SIGN are normally expressed on cell surfaces as multimers (reported as trimers and tetramers, respectively) with intertwined C-type lectin domains held together by an α-helical coiled-coil stalk^26^. In order to mimic this native configuration, we generated human embryonic kidney (HEK) 293 Freestyle cells stably expressing either human CD23 or DC-SIGN. FcγRIIa (CD32a)-expressing HEK cells were generated and used as positive control for low affinity IgG binding. FACS results showed that human FcγRIIa, CD23 and DC-SIGN were specifically recognized by antibodies directed against the corresponding receptor (Fig. 2a–c and Supplemental Fig. S2 for representative histograms). To verify the functionality of each receptor, we tested the binding to their natural ligands (IgG1 for FcγRIIa; IgE for CD23; ICAM-3 for DC-SIGN) in FACS, and found that each receptor specifically recognized and bound their ligand. (Fig. 2a–c and Supplemental Fig. S2 for representative histograms).

### Soluble human IgG1 glycoforms do not bind to CD23- or DC-SIGN-expressing cells

In order to investigate binding to human IgG1, we then tested binding of the IgG1 glycoforms to the cells by FACS, in the presence of calcium. No binding was observed at 37 °C for cells expressing either CD23 or DC-SIGN, not even for the Fab-sialylated or the most prevailing naturally-occurring sialylated Fc-glycoform (+F−B) (Fig. 2d and Supplemental Fig. S2 for representative histograms). Also, a lower binding temperature (4 °C) or lower IgG1 concentrations did not result in binding (Supplemental Fig. S2). In contrast, substantial binding was observed for all Fc- and Fab-glycosylated IgG1 to the low affinity FcγRIIa.

### CD23/DC-SIGN-expressing cells do not bind to immobilized IgG1 glycoforms

**Figure 3:**
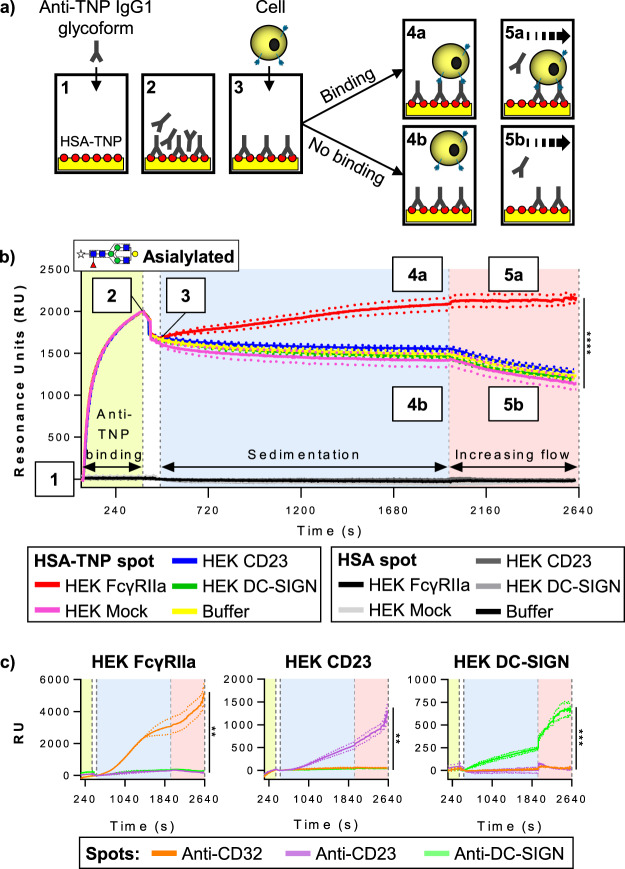
Cellular SPR imaging layout and detection of receptor expression. (**a**) Schematic layout of the experimental setup for the cellular SPR, with HSA-TNP antigens depicted as red circles, SPR-sensor as yellow block, receptor-expressing cells as yellow circles with blue receptors and increasing gradient flow with a dashed arrow. (**b**) Representative sensorgram of five cellular SPRi runs on HSA-TNP- (10 nM) and HSA- (100 nM) (negative control) coated spots. Numbers depicted in the sensorgram correspond to the schematic representations in A, which shows the processes on the HSA-TNP spots at different time points. 1 IgG1 glycoform (asialylated in this case) injection; 2 Anti-TNP glycoform binding to HSA-TNP spot; 3 Receptor-bearing cell injection; 4 and 5 Cell sedimentation phase and start gradient flow respectively. 4a, 5a show cell binding and 4b, 5b no binding scenario. Green, blue and red backgrounds in the sensorgram represent the anti-TNP glycoform immobilization, cell sedimentation and gradient flow (1–120 µl/s) phase, respectively. Each line represents the binding of a specific cell type (HEK FcγRIIa in red, HEK Mock in pink, HEK CD23 in blue, HEK DC-SIGN in green for the HSA-TNP spots) in resonance units (RU) to immobilized asialylated anti-TNP IgG1 through time, with buffer flow (in yellow) to determine the background signal. (**c**) Representative sensorgrams show the binding of HEK FcγRIIa, HEK CD23 and HEK DC-SIGN cells to spots coated with murine antibodies against CD32, CD23 and DC-SIGN, respectively. These spots functioned as cell flow controls to confirm the presence of receptors. Significant differences compared to control (HEK Mock (panel B) or isotype control spots (panel C)) were based on the RU values on the last time point of the flow phase, determined by One-way ANOVA with Tukey’s multiple comparisons test (**P < 0.01, ***P < 0.001, ****P < 0.0001). Data are representative of three independent experiments showing mean ± standard deviation (s.d.). Each line represents the average sensorgram, with s.d. as dotted lines, from at least three spots monitored in real time simultaneously.

**Figure 4:**
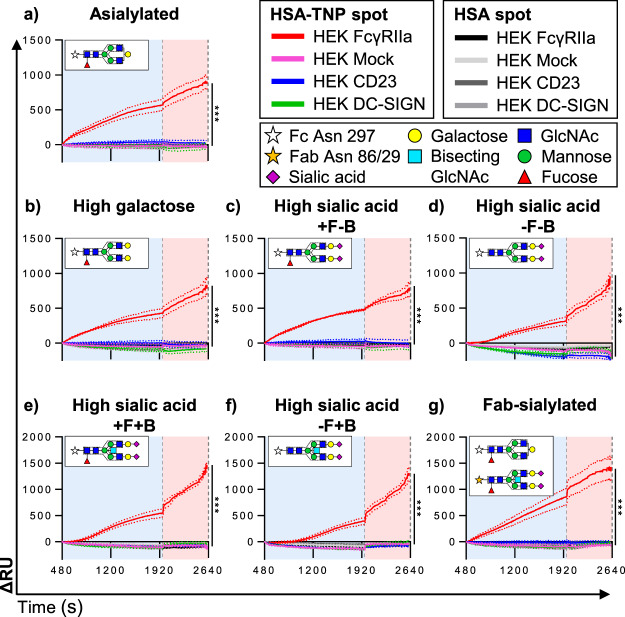
Neither human CD23- nor DC-SIGN-expressing cells bind human IgG1-containing immune complexes, regardless of glycosylation status. Cell flow sensorgrams after spot-specific buffer flow (background) subtraction (ΔRU). (**a**–**g**) Sensorgrams show binding responses to the different HSA-TNP (10 nM)-immobilized IgG1 glycoforms (specific glycan depicted in inset) of HEK DC-SIGN and HEK CD23 compared to HEK Mock with HEK FcγRIIa as positive control. Line identities and sugar moieties are represented by different shapes as indicated in the figure legend. Data are representative of three independent experiments showing mean ± s.d. Each line represents the average sensorgram, with s.d. as dotted lines, from at least three spots monitored in real time simultaneously. Significant differences compared to HEK Mock were based on the RU values on the last time point of the flow phase, determined by One-way ANOVA with Tukey’s multiple comparisons test (***P < 0.001).

Although the low affinity binding of human CD23 to IgE (Fig. 2b and Supplemental Fig. S2) and ICAM-3 binding to DC-SIGN (Fig. 2c and Supplemental Fig. S2) was detected by FACS, we could not rule out that even lower affinity interactions were not being detected in these assays. Therefore, and to study potential avidity-dependent interactions between human IgG1 and CD23/DC-SIGN, cellular Surface Plasmon Resonance imaging (cSPRi) tests were performed. Here we used trinitrophenyl-conjugated human serum albumin (HSA-TNP) as a model antigen which was spotted onto the sensor. Anti-TNP IgG1 glycoforms were injected and binding and formation of antigen-antibody complexes was observed in real-time (Fig. 3a; 1–2). This was followed by the injection of the generated cell lines (Fig. 3a; 3). First, without flow, the cells were allowed to sediment and interact with the antigen-bound antibodies in the presence of calcium (Fig. 3a; 4). Subsequently, the immune-complex-bound cells were washed with increasing flow speed (Fig. 3a; 5). Importantly, none of the cell lines showed binding to the negative control spots (Fig. 3b). Compared to Mock-transfected cells, FcγRIIa-expressing HEK cells bound asialylated IgG1 and, as expected, no binding was observed for Mock-, CD23-, or DC-SIGN-expressing HEK cells (Fig. 3b). Both CD23- and DC-SIGN-expressing cells showed an increased binding response on spotted anti-CD23 and anti-DC-SIGN, respectively (Fig. 3c). Similarly, the FcγRIIa-expressing HEK cells showed binding to anti-CD32 on the SPRi-sensor.

Importantly, no significant binding of CD23-, DC-SIGN-, or Mock-expressing HEK cells was observed to human IgG1 with varying degrees of Fc-glycan sialylation (Fig. 4a–c), irrespective of the presence or absence of fucose and/or bisecting GlcNAc moieties (Fig. 4c–f). Moreover, the presence of sialylated Fab-glycans did not trigger the binding of any of these cells (Fig. 4g). In addition, varying the HSA-TNP antigen density level either 10 times higher or lower (100 and 1 nM) did not show specific binding of CD23/DC-SIGN-expressing cells to Fc- or Fab-sialylated IgG1 (Supplemental Fig. S3).

## Discussion

In this study, we found no evidence that human IgG1 binds either native CD23 or DC-SIGN regardless of the sialylation status of IgG, whereas binding of these receptors to their native ligands was easily detected. These data are, however, in full agreement with those of Crispin *et al*. who also found no binding of sialylated IgG Fc to DC-SIGN^30,40^. Here, we investigated the binding in more detail by using a biosensor platform, which allowed us to study avidity interactions, and the study was extended by including CD23. In addition, the context of the IgG sialylation was studied extensively, by including both Fab-sialylated IgG and altering the naturally-occurring variable glycan adducts (fucose and bisecting GlcNAc) in the biantennary sialylated Fc-glycan, in any possible combination. Still no binding could be detected, while binding was detected for the low affinity FcγRIIa which acted as a positive control. Taken together, these data do not support CD23 and DC-SIGN to be classified as true FcγRs.

Part of the explanations for the opposing findings here and in literature concerning the binding of IgG to either CD23 and/or DC-SIGN^14,17–19,21,23,25,30,31,33,40,42^ may be due to differences in methodology and materials. For instance, most previous studies lack a characterization of the glycoforms used and relied on SNA-purified IgG from serum or IVIg, or *ex vivo* treatment of IgG or fragments thereof. Differences in used disease models may also be a reason for the apparent contradictions in literature. Many of these studies used human IgG in a murine context, which also introduces slight differences in structural composition of sialic acid between human and mice^49–52^. Other studies demonstrating binding of sialylated human IgG to DC-SIGN- or CD23-expressing CHO cells^18,23^, possibly introducing an incomplete human context e.g. the lack of α2,6-linked sialic acid, addition of alpha-Gal, and perhaps elevated level of mannose-containing structures^52,53^. In addition, in most *in vivo* studies human IgG was administered to mice which express SIGN-R1, potentially resulting in an incorrect translation from murine to human setting^18,21,33,41^.

Considerable part of the evidence for the binding between CD23/DC-SIGN to sialylated human IgG is based on predicted interactions between crystal structures. The structure of the sialylated human IgG1 Fc domain was determined by Ahmed *et al*. and showed that upon sialylation the Fc domain indeed undergoes a conformational change in the CE loop of the CH2 domain resulting in an increased interdomain flexibility^29^. However, no considerable conformational differences can be observed in the IgG1 CH2-CH3 interphase where CD23 and DC-SIGN have been proposed to bind^23^. The group of Crispin *et al*. also determined the structure of the human sialylated Fc domain and was not able to observe conformational changes upon sialylation, even not in the CH2 domains^30^. Within the same group, Yu *et al*. demonstrated that there is no specific binding of IgG to DC-SIGN tetramers, regardless of sialylation status, and that serum IgG was not able to inhibit the binding of DC-SIGN to natural ligand HIV envelope glycoprotein gp120^40^. Therefore, these authors concluded that the direct binding between DC-SIGN and human IgG is uncertain. Another aspect of the proposed sialylation-induced “closed” IgG Fc state, is the major reduction (up to 10-fold) in binding affinity of IgG1 to conventional FcγRs^18,22^. Currently, evidence is growing that IgG Fc-sialylation does not or only subtly affect binding affinities to FcγRs^54,55^. While Subedi and Barb found a modest increase in affinity (1.16-fold) for afucosylated IgG1 to both FcγRIIa and FcγRIIIa/b^55^, our group previously found a slight decrease (approximately 2-fold) in affinity of whole IgG1 for FcγRIIIa/b due to sialylation, but only when the Fc-glycan was also afucosylated and bisected^7^.

Our results do not imply that the function of IgG1 (or other subclasses) Fc-/Fab-sialylation or the involvement of C-type lectins (or other lectins, e.g. CD22) in IVIg therapy can be completely excluded. The mode of action of IVIg is complex, involving many underlying mechanisms that are both Fab- and Fc-mediated^14,22^. The most important mode of action is likely depending on its context. However, we conclude that a direct binding of either CD23 or DC-SIGN to sialylated IgG is very unlikely to occur. Therefore, these molecules should not be considered as *bona fide* receptors for IgG in humans.

## Methods

### Cloning

DNA encoding human DC-SIGN (AAK20997.1) was synthesized and codon-optimized by Geneart (Invitrogen, USA) and DNA encoding human CD23 cDNA (BC062591) and FcγRIIa 131 H (XM_017000664.1) was synthesized by Integrated DNA Technologies, USA. All constructs were generated with flanking HindIII and EcoRI restriction sites for cloning into pcDNA3.1 (+) expression vector (Invitrogen, USA) with ampicillin and neomycin resistance genes using Rapid Ligation Kit (Thermo Scientific, USA). Ligation products were transformed into DH5α heat-shock competent cells (Thermo Scientific, USA). DNA sequences isolated from Ampicillin resistant clones were verified by Sanger sequencing in a 3730 DNA Analyzer.

### Stable cell line production

HEK 293 FreeStyle cells were transfected with linearized empty, DC-SIGN, CD23 or FcγRIIa 131 H pcDNA3.1 expression vectors using 293fectin (Invitrogen, USA). These cell lines were cultured in suspension at 37 °C, 8% CO_2_ on a shaker in serum-free FreeStyle 293 Expression Medium (Gibco Life Technologies, USA) with 250 µg/ml Geneticin/G418 (PAA the cell culture company, Austria) added as selection marker. Receptor surface expression was monitored by flow cytometry.

### Antibodies and other reagents

Human anti-TNP IgG1 production and glyco-engineering was performed as described by Dekkers *et al*. for Fc-glycoforms^8,43^. In brief, vectors encoding p21, p27 and pSVLT and a single-gene construct encoding human anti-TNP IgG1 heavy chain and kappa light chain were transiently transfected into HEK 293 FreeStyle cells using 293fectin (Invitrogen, USA). To add bisecting GlcNAc to the N297 glycan, cells were co-transfected with a vector encoding GlcNAc transferase (GNTIII). To decrease fucosylation, decoy substrate 2-fluorofucose (2FF) was added to the medium. For IgG1 galactosylation, D-galactose (Sigma-Aldrich Chemie N.V., The Netherlands) was added to the medium and cells were co-transfected with a vector encoding beta-1,4-galactosyltransferase (B4GALT1). For α2,6-sialylation, the latter treatment was combined with co-transfection with a vector encoding beta-galactoside alpha-2,6-sialyltransferase 1 (ST6GALT) and after purification these antibodies were further sialylated *in vitro* by incubation with recombinant ST6GALT enzyme and the substrate cytidine-5′-monophospho-*N*-acetylneuraminic acid. Unmodified anti-TNP IgG1 was used as a control for asialylation. For the Fab-glycoform, glycosylation sites in the VH and VL domain were introduced by substituting respectively the serine residue on position 29 and aspartic acid residue on position 86 into asparagine. Constructs encoding the anti-TNP IgG1 heavy chain- (S29N) and kappa light chain- (D86N) Fab glycosylation were transfected into HEK cells in the same manner as the anti-TNP Fc-glycoforms. For all glycoforms, cell supernatant was harvested five days after transfection and purified using a HiTrap Protein A HP column (GE Healthcare, USA) on an ÄKTAprime plus system (GE Healthcare, USA). Commercially available antibodies and other reagents used in ELISA, FACS and cSPRi tests are depicted in Table 1.

**Table 1:** Overview of the used commercial antibodies and other reagents.

Name	Conjugate	Clone	Firm	Application
Mouse anti CD32	Biotin	AT10	Bio-Rad	F
Mouse anti-CD32	n.a.	AT10	Genetex	C
Mouse anti-CD23	PE	EBVCS-5	BD Biosciences	F, C
Mouse anti-CD23	n.a.	BU38	Ancell Corporation	C
Mouse anti-CD209	PE	MR-1	Bio-Rad	F, C
Mouse anti-DC-SIGN	n.a.	MR-1	Bio-Rad	C
Human IgE	APC*	n.a.	Abcam	F
ICAM-3 His-tagged	n.a.	n.a.	Sino Biological	F
Mouse anti-His tag	FITC	6G2A9	Genscript	F
Mouse IgG1κ isotype	PE	203	Sanquin	F, C
Mouse IgG1κ isotype	n.a.	203	Sanquin	C
Goat anti-human IgG1 F(ab’)_2_	PE	n.a.	Southern Biotech	F
Streptavidin	APC	n.a.	Biolegend	F, E
Streptavidin	HRP	n.a.	Sanquin	E
Mouse anti-human IgG	n.a.	MH16-1	Sanquin	E
Mouse anti-human IgG	HRP	MH16-1	Sanquin	E
*Sambucus nigra* agglutinin	Biotin	n.a.	Elderberry Bark lectin, Vector Labs	E

Abbreviations: F = FACS, C = cellular SPRi, E = ELISA, n.a. = not applicable.

*Labeled with APC using LYNX kit (Bio-Rad, USA) according to the manufacturer’s protocol.

### Trypsin digestion and mass spectrometric glycosylation analysis

The IgG samples were dried in a centrifugal vacuum concentrator (Eppendorf, Germany) for 2 hours at 60 °C. Subsequently, the IgG was digested overnight at 37 °C in 40 µl (25 mM) ammonium bicarbonate (Sigma-Aldrich Chemie N.V., The Netherlands) containing 200 ng trypsin (sequencing grade, Promega, Madison, WI). The resulting glycopeptides were analyzed by nanoLC-ESI-QTOF-MS on an RSLCnano Ultimate 3000 system (Thermo Fisher, The Netherlands) coupled to an Impact quadrupole-TOF-MS (micrOTOF-Q, Bruker Daltonics, Germany). The system and glycopeptide analysis are described in detail by Dekkers *et al*.^43^.

### Lectin enzyme-linked immuno sorbent assay

To confirm the occupancy of the Fab glycosylation sites, lectin ELISA was performed as described previously by van de Bovenkamp *et al*.^48^. In brief, 96-well flat bottom plates (Nunc MaxiSorp, Denmark) were coated overnight with neuraminidase (New England Biolabs, USA)-treated mouse anti-human IgG diluted in PBS. Plates were incubated for 1 hour with 100 µl sample (25 ng/ml) diluted in PBS supplemented with 0.02% Tween-20. Subsequently, plates were incubated with biotinylated *Sambucus nigra* agglutinin (SNA) for 2 hours for sialylation measurement. Then, plates were incubated with HRP-conjugated streptavidin for 10 minutes. In order to correct sialylation optical densities for total IgG, in parallel, total IgG levels were determined for all samples by incubating for 1 hour with HRP-conjugated mouse anti-human IgG. Plates were washed three times in between each incubation step and detection was performed with tetramethylbenzidine and stopped with 2 M H_2_SO_4_. Absorbance was measured at 450 and 540 nm on a BioTek plate reader.

### Flow cytometry

HEK DC-SIGN/CD23/FcγRIIa/Mock-transfected HEK293 (1.5 ∙ 10^5^ cells per well) were incubated, stained and washed in 20 mM HEPES (Sigma-Aldrich Chemie N.V., The Netherlands) buffer supplemented with 1 mM CaCl_2_ (Merck Chemicals B.V., The Netherlands), 1 mM MgCl_2_ (Merck Chemicals B.V., The Netherlands) and 1% BSA (Sigma-Aldrich Chemie N.V., The Netherlands), pH 7.4. After washing, directly-labeled samples were incubated with antibodies against the expressed receptor or natural ligand. Indirectly-labeled samples were incubated with either recombinant His-tagged human ICAM-3, human anti-TNP IgG1 glycoforms or biotinylated mouse anti-CD32 and subsequently incubated with fluorescently labeled secondary antibodies/F(ab’)_2_ fragments or streptavidin. Incubation times were one hour and all steps were performed at 4 °C, except for the ligand and IgG glycoform 37 °C binding studies. Sample fluorescence was measured by a FacsCanto II cytometer (BD Biosciences, USA) and at least 10,000 events were recorded per sample. Reported data show geometric mean fluorescence intensities with the unstained cell signals (autofluorescence) subtracted.

### Cellular surface plasmon resonance imaging

An Easy2Spot pre-activated SensEye P-type sensor (Ssens, The Netherlands) was spotted, in triplicate, with HSA-TNP^7^ (100, 10, 1 nM), HSA (100 nM) (Sanquin, The Netherlands) and antibodies against CD23/DC-SIGN/CD32 (100 nM) by a continuous flow microspotter (CFM) (Wasatch Microfluidics, USA). Spotting buffer consisted of 10 mM sodium acetate/acetic acid supplemented with 0.075% Tween-80 at pH 5.5, except for HSA and HSA-TNP, which was pH 4.0. Deactivation of the sensor was performed with 100 mM ethanolamine, pH 8.0 supplemented with 0.075% Tween-80 and 0.1% HSA in acetate buffer, pH 3.7 supplemented with 0.075% Tween-80. Analyte binding was performed in an IBIS MX96 (IBIS Technologies B.V., The Netherlands). After injection, antibodies were allowed to bind to HSA-TNP spots for 5 minutes. A short wash was performed to remove the unbound antibodies from the sensor. Subsequently, cells were injected and allowed to sediment/bind to immobilized glycoforms or control spots for 25 minutes^56^. During the next 11 minutes, the wash flow speed was increased stepwise from 1 to 120 µl/s. Regeneration was performed using glycine HCl pH 2.2 supplemented with 0.075% Tween-80.

In the first (antibody) flow either one of the human IgG1 glycoforms (100 nM) was injected into the flow chamber, in the second (cell) flow either HEK DC-SIGN/CD23/FcγRIIa/Mock (5.0 ∙ 10^6^ cells per ml). System buffer consisted of 20 mM HEPES buffer supplemented with 1 mM CaCl_2_, 1 mM MgCl_2_ and 1% BSA, pH 7.4.

### Data analysis

FACS data was analyzed using FlowJo software (FlowJo, USA). cSPRi data was analyzed using SprintX software (IBIS Technologies B.V., The Netherlands) and Rstudio software (RStudio, USA)^57^. Graphpad Prism 8.0.2 (GraphPad Software, Inc., USA) was used to generate sensorgrams and to perform statistical analyses. To optimize visualization of the cell binding during sedimentation and flow phase in the cSPRi data, per HSA-TNP density, consecutively individual sensorgrams were normalized to the representative antibody binding plateau and buffer signals were spot-specifically subtracted for each time point.

## Supplementary information

Supplementary Information

## Data Availability

We will make materials, data and associated protocols available to readers.
