# Author Correction: Human DC-SIGN and CD23 do not interact with human IgG

**DOI:** 10.1038/s41598-020-68760-2

**Published:** 2020-07-23

**Authors:** A. Robin Temming, Gillian Dekkers, Fleur S. van de Bovenkamp, H. Rosina Plomp, Arthur E. H. Bentlage, Zoltán Szittner, Ninotska I. L. Derksen, Manfred Wuhrer, Theo Rispens, Gestur Vidarsson

**Affiliations:** 1 Department Experimental Immunohematology, Sanquin Research and Landsteiner Laboratory, Academic Medical Centre, University of Amsterdam, Amsterdam, The Netherlands; 2 Department Immunopathology, Sanquin Research and Landsteiner Laboratory, Academic Medical Centre, University of Amsterdam, Amsterdam, The Netherlands; 3 Center for Proteomics and Metabolomics, Leiden University Medical Center, Leiden, The Netherlands

Correction to: *Scientific Reports* 10.1038/s41598-019-46484-2, published online 10 July 2019

The original version of this Article contained errors in the Reference list. Reference 26 was incorrectly listed as reference 48, and reference 27 was incorrectly listed as reference 31. As a result, the original references 26–30 are now listed as references 28–32, and references 32–47 are now listed as references 33–48. This has been corrected in both the HTML and PDF versions of this Article.

