# Supplementary material for: Human DC-SIGN and CD23 do not interact with human IgG
Source: Sci Rep. 2019 Jul 10;9:9995. doi: 10.1038/s41598-019-46484-2 (PMC6620288; doi:10.1038/s41598-019-46484-2)
Supplement: Supplementary file 1 — Supplementary Information [file 41598_2019_46484_MOESM1_ESM.pdf]

Supplementary information

Human DC-SIGN and CD23 do not interact with human IgG

A.R. Temming[1], G. Dekkers[1], F.S. van de Bovenkamp[2], H.R. Plomp[3], A.E.H. Bentlage[1], Z. Szittner[1], N.I.L. Derksen[2], M. Wuhrer[3], T. Rispens[2], G. Vidarsson[1,*]

[1] Department Experimental Immunohematology, Sanquin Research and Landsteiner Laboratory, Academic Medical Centre, University of Amsterdam, Amsterdam, The Netherlands

[2] Department Immunopathology, Sanquin Research and Landsteiner Laboratory, Academic Medical Centre, University of Amsterdam, Amsterdam, The Netherlands

[3] Center for Proteomics and Metabolomics, Leiden University Medical Center, Leiden, The Netherlands

[*] Corresponding author:
Gestur Vidarsson
G.Vidarsson@sanquin.nl
Plesmanlaan 125
1066CX Amsterdam
The Netherlands
Tel: 0031 20512 3377
Fax: 0031 20512 3474

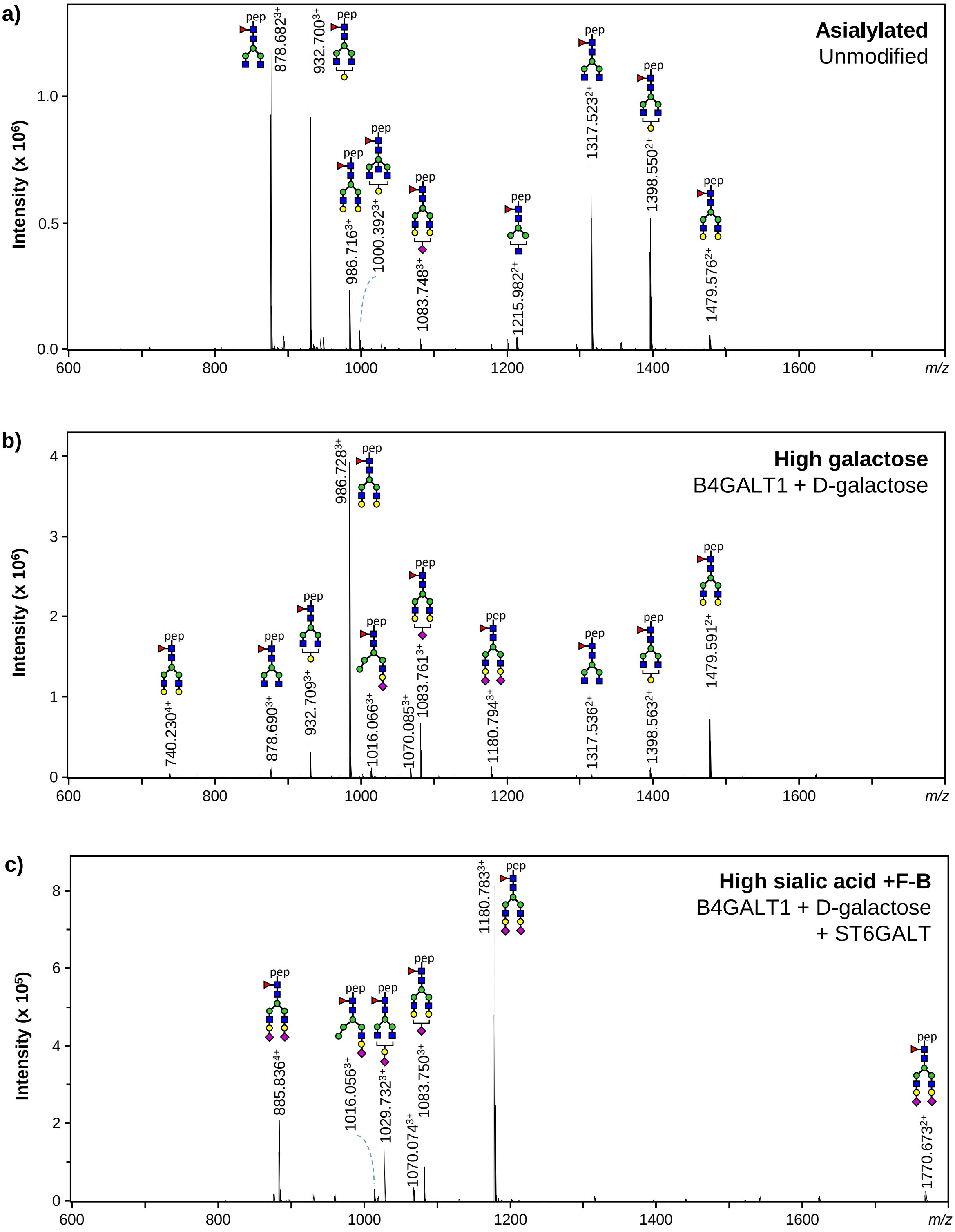
a)
Asialylated
Unmodified
Intensity (x 10⁶)
878.682³⁺
932.700³⁺
986.716³⁺
1000.392³⁺
1083.748³⁺
1215.982²⁺
1317.523²⁺
1398.550²⁺
1479.576²⁺
pep
m/z
b)
High galactose
B4GALT1 + D-galactose
Intensity (x 10⁶)
740.230⁴⁺
878.690³⁺
932.709³⁺
986.728³⁺
1016.066³⁺
1070.085³⁺
1083.761³⁺
1180.794³⁺
1317.536²⁺
1398.563²⁺
1479.591²⁺
pep
m/z
c)
High sialic acid +F-B
B4GALT1 + D-galactose
+ ST6GALT
Intensity (x 10⁵)
885.836⁴⁺
1016.056³⁺
1029.732³⁺
1070.074³⁺
1083.750³⁺
1180.783³⁺
1770.673²⁺
pep
m/z

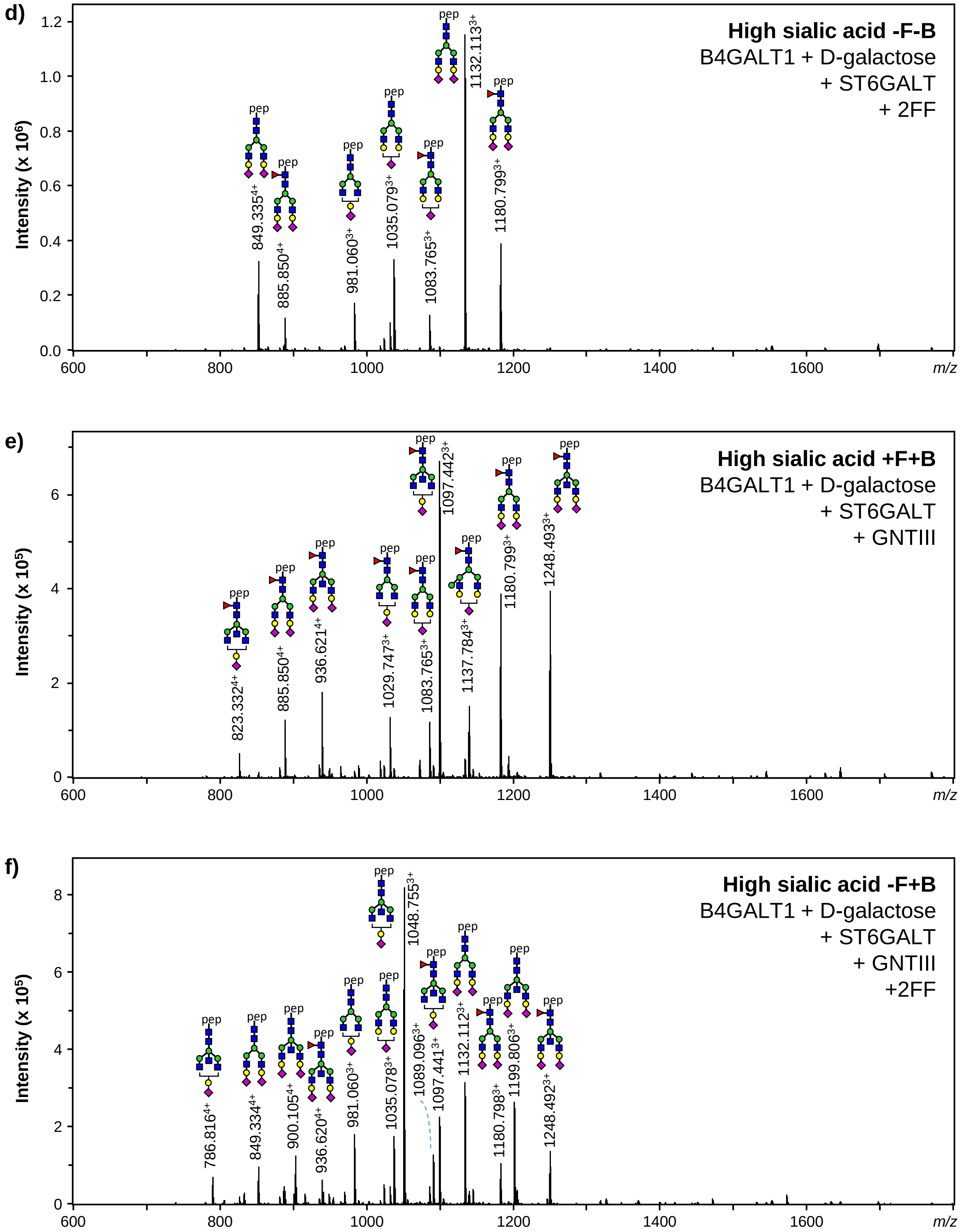

Supplemental Figure S1: *N*-glycan profiles of glyco-engineered anti-TNP Fc-glycoforms
A-F) Mass spectra of IgG Fc glycopeptides. Glyco-engineering treatments are depicted under the glycoform name. *N*-glycan structures are schematically represented; galactose, yellow circle; mannose, green circle; *N*-acetylglucosamine, blue square; fucose, red triangle; sialic acid, purple diamond; pep, peptide moiety of the IgG1 Fc glycopeptide.

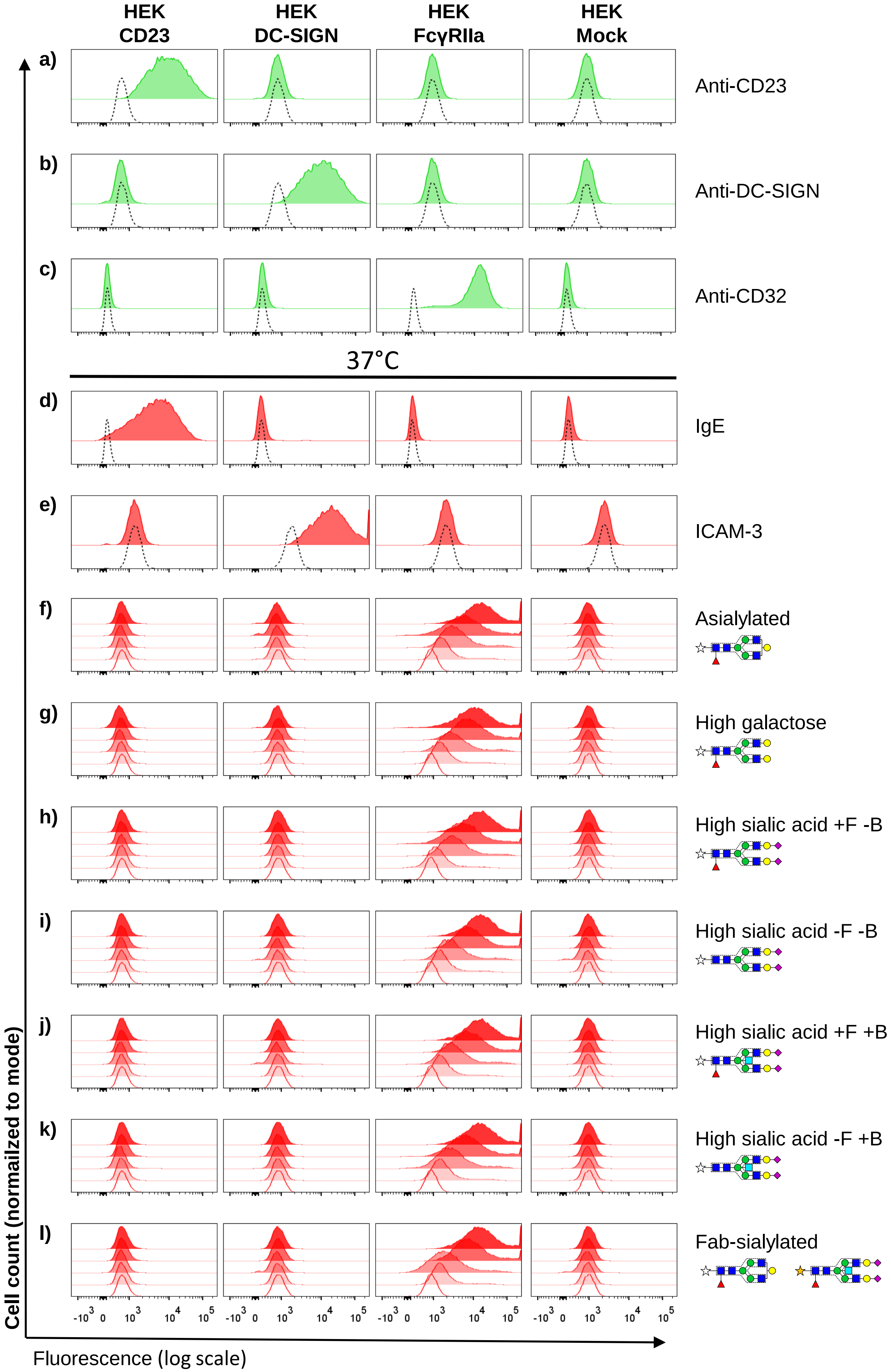
HEK CD23
HEK DC-SIGN
HEK FcγRIIa
HEK Mock
a)
b)
c)
d)
e)
f)
g)
h)
i)
j)
k)
l)
Anti-CD23
Anti-DC-SIGN
Anti-CD32
37°C
IgE
ICAM-3
Asialylated
High galactose
High sialic acid +F -B
High sialic acid -F -B
High sialic acid +F +B
High sialic acid -F +B
Fab-sialylated
-10³ 0 10³ 10⁴ 10⁵
Cell count (normalized to mode)
Fluorescence (log scale)

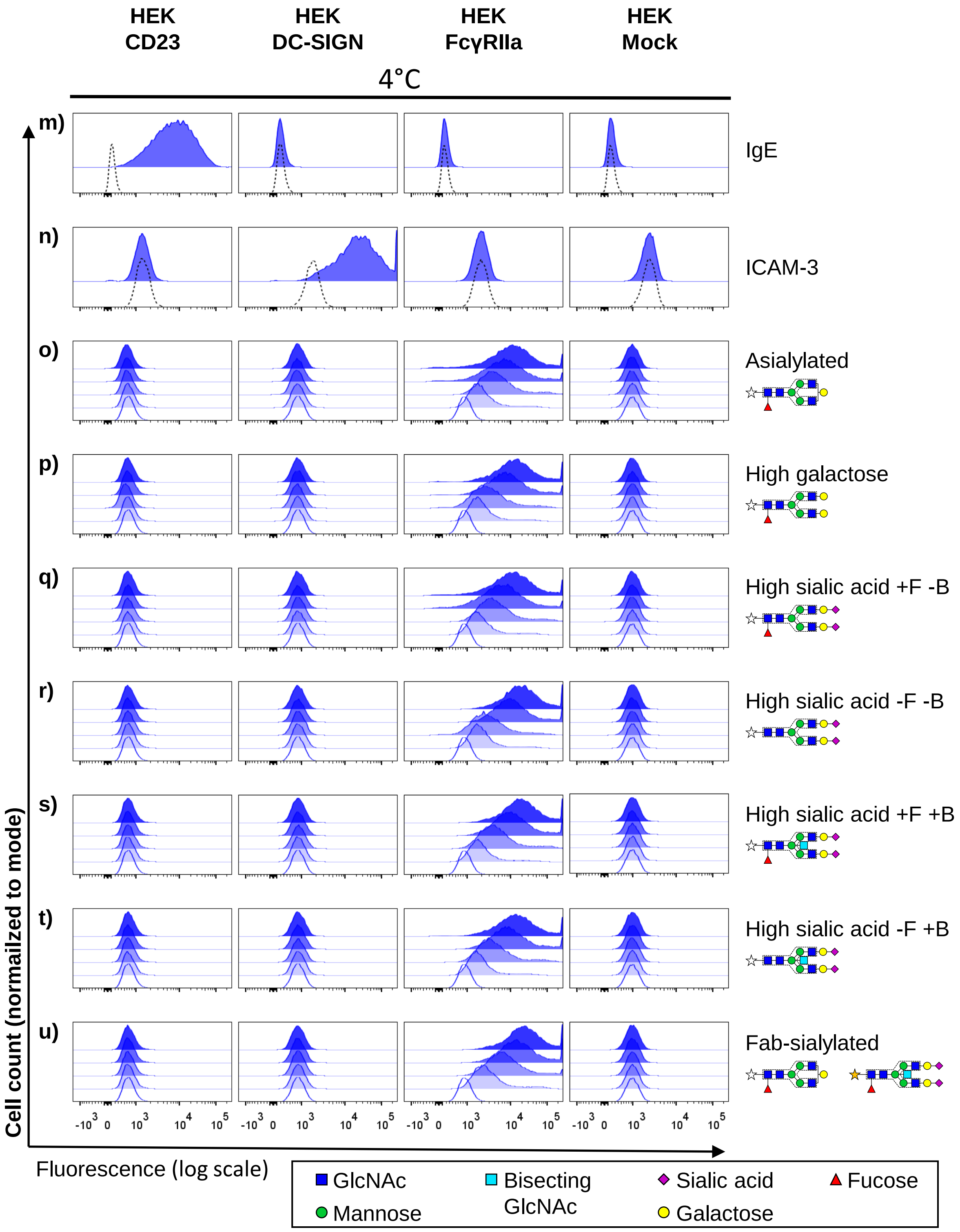

Supplemental Figure S2: FACS histograms of HEK CD23/DC-SIGN/FcγRIIa/Mock cells for receptor expression and binding to ligand and IgG1 glycoform

Representative FACS histograms (cell counts normalized to mode) showing binding of receptor-expressing HEK cell lines to antibodies directed against their receptor (A-C in green), specific ligand (D-F at 37°C in red, and M-O at 4°C in blue) or IgG1 Fc-/Fab-glycoforms (F-L at 37°C in red, and O-U at 4°C in blue) at antibody concentrations of 10, 3, 1, 0.3 and 0 µg/ml (from high to low color intensity, respectively). Black-dashed transparent histograms represent isotype control (mouse IgG1 for anti-DC-SIGN, anti-CD23), secondary only (anti-His for His-tagged human ICAM-3, streptavidin for biotinylated anti-CD32) or no stain (for IgE). Data are representative of three independent experiments.

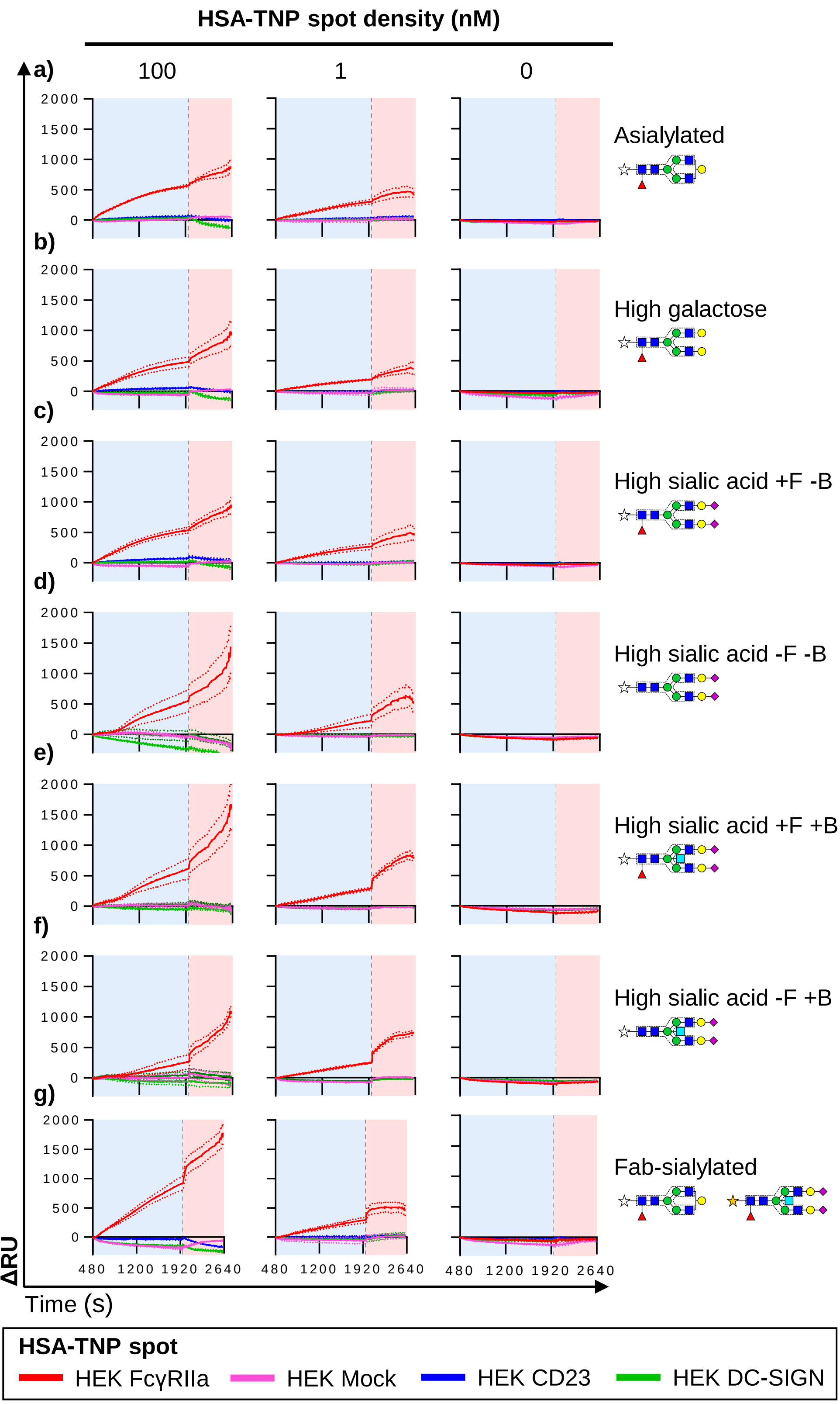

Supplemental Figure S3: Buffer-blanked cell flow sensorgrams of HEK CD23/DC-SIGN/FcγRIIa/Mock for immobilization concentrations 100, 1 and 0 nM for all different IgG1 glycoforms

Cell flow sensorgrams after spot-specific buffer flow (background) subtraction (ΔRU). A-G) Sensorgrams show no avidity-dependency of HEK CD23 and HEK DC-SIGN for the binding to seven different HSA-TNP-immobilized IgG1 glycoforms compared to HEK Mock. HEK FcγRIIa positive control cells show binding to all glycoforms. The start of stepwise increasing flow is indicated by the grey dashed line. The 0 nM HSA-TNP spot was coated with 100 nM HSA. Data are representative of at least two independent experiments showing mean ± s.d. Each line represents the average sensorgram, with s.d. in dotted lines, from at least three spots monitored in real time and simultaneously.
